# Site-Specific Fat-1 Knock-In Enables Significant Decrease of n-6PUFAs/n-3PUFAs Ratio in Pigs

**DOI:** 10.1534/g3.118.200114

**Published:** 2018-03-21

**Authors:** Mengjing Li, Hongsheng Ouyang, Hongming Yuan, Jianing Li, Zicong Xie, Kankan Wang, Tingting Yu, Minghao Liu, Xue Chen, Xiaochun Tang, Huping Jiao, Daxin Pang

**Affiliations:** Jilin Provincial Key Laboratory of Animal Embryo Engineering, College of Animal Sciences, Jilin University, Changchun, Jilin Province, People’s Republic of China

**Keywords:** pig, *fat-1*, genome editing, omega-3 polyunsaturated fatty acids

## Abstract

The *fat-1* gene from *Caenorhabditis elegans* encodes a fatty acid desaturase which was widely studied due to its beneficial function of converting n-6 polyunsaturated fatty acids (n-6PUFAs) to n-3 polyunsaturated fatty acids (n-3PUFAs). To date, many *fat-1* transgenic animals have been generated to study disease pathogenesis or improve meat quality. However, all of them were generated using a random integration method with variable transgene expression levels and the introduction of selectable marker genes often raise biosafety concern. To this end, we aimed to generate marker-free *fat-1* transgenic pigs in a site-specific manner. The Rosa26 locus, first found in mouse embryonic stem cells, has become one of the most common sites for inserting transgenes due to its safe and ubiquitous expression. In our study, the *fat-1* gene was inserted into porcine Rosa 26 (pRosa26) locus via Clustered Regularly Interspaced Short Palindromic Repeats (CRISPR)/CRISPR-associated 9 (Cas9) system. The Southern blot analysis of our knock-in pigs indicated a single copy of the *fat-1* gene at the pRosa26 locus. Furthermore, this single-copy *fat-1* gene supported satisfactory expression in a variety of tissues in F1 generation pigs. Importantly, the gas chromatography analysis indicated that these *fat-1* knock-in pigs exhibited a significant increase in the level of n-3PUFAs, leading to an obvious decrease in the n-6PUFAs/n-3PUFAs ratio from 9.36 to 2.12 (***P < 0.0001). Altogether, our *fat-1* knock-in pigs hold great promise for improving the nutritional value of pork and serving as an animal model to investigate therapeutic effects of n-3PUFAs on various diseases.

The n-6PUFAs and n-3PUFAs have an important role in regulating biological processes. The n-3PUFAs include alpha-linolenic acid (ALA), eicosapentaenoic acid (EPA), docosahexaenoic acid (DHA) and docosapentaenoate acid (DPA). The n-6PUFAs mainly include arachidonic acid (AA) and linoleic acid (LA) (Kim *et al.* 2016; Zhu *et al.* 2008). N-3PUFAs provide broad potential health benefits according to previous studies, and thus are increasingly considered as necessary nutrients in a daily diet (Sioutis *et al.* 2008). It has been showed that cancer cells rich in n-3PUFAs underwent apoptosis whereas control cancer cells proliferated normally (Kim *et al.* 2018; Ge *et al.* 2002). Furthermore, a lower n-6PUFAs/n-3PUFAs ratio diet could attenuate disease progression of depressive patients compared to the control group (Beydoun *et al.* 2015). Recent study provided new insights to the protective effect of n-3 PUFAs against non-alcoholic liver disease (McCormick *et al.* 2015). Therefore, a lower n-6PUFAs/n-3PUFAs ratio is particularly important for a healthy diet. However, the lack of an enzyme capable of converting n-6PUFAs into n-3PUFAs leads to a high n-6PUFAs/n-3PUFAs ratio in mammals. It has consistently been considered unhealthy when excessive mammalian meat products were included in modern diet (Cheng *et al.* 2015; Lai *et al.* 2006).

The *fat-1* gene from *Caenorhabditis elegans* provided a feasible solution to the abovementioned problem because the fatty acid desaturase,it encoded, is capable of converting n-6PUFAs to n-3PUFAs by joining a double bond at the n-3 hydrocarbon position of the n-6PUFAs (Kang 2005). Researchers have developed *fat-1* transgenic mouse (Kang *et al.* 2004), pig (Lai *et al.* 2006) and cow (Wu *et al.* 2012) in which the n-6PUFAs were successfully converted into n-3PUFAs. Furthermore, these transgenic animal models have been used to study a wide range of diseases such as arthritis (Woo *et al.* 2015), allergic reactions (Bilal *et al.* 2011), cardiovascular diseases (Kris-Etherton *et al.* 2004), cancers (Han *et al.* 2016a) and Alzheimer’s disease (Wu *et al.* 2016a). Indeed, these *fat-1* transgenic animals that are rich in n-3 PUFAs alleviate these diseases at different levels. However, all reported *fat-1* transgenic animals were obtained by random integration, which may lead to variable expression levels and unstable phenotypes due to multiple-copy integration and uncontrollable insertion sites. Instead, site-specific integration allows the exogenous gene to be inserted into a specific locus, enables stable expression of transgenes at defined sites (Nandy and Srivastava 2011; Obayashi *et al.* 2012). Furthermore, *fat-1* transgenic animals generated by conventional transgenic methods carry selectable marker genes that may disrupt the expression of endogenous genes and increase public concern regarding the release of antibiotic genes into the environment (Yu *et al.* 2013). Hence, generating selectable marker-free as well as site-specific transgenic animals could ease the potential biosafety problem.

The Rosa26 gene was originally found in mouse embryonic stem cells and subsequently identified in human, rat, pig, sheep and rabbit (Friedrich and Soriano 1991; Irion *et al.* 2007; Kobayashi *et al.* 2012; Li *et al.* 2014; Wu *et al.* 2016b; Yang *et al.* 2016). It directs ubiquitous expression of a non-coding RNA in embryonic and adult tissues (Kong *et al.* 2014). In addition, this locus was proved to be a safe harbor because the transgenes inserted here do not interfere with the function of endogenous genes in mice (Friedrich and Soriano 1991). At present, many Rosa26 targeted animals have been successfully generated. Accordingly, we considered inserting *fat-1* gene at this locus so as to achieve controllable expression under the control of endogenous Rosa26 promoter. As a powerful and widely used tool, CRISPR/Cas9 could efficiently produce flexible genome modifications, including deletion, insertion (Wu *et al.* 2016b; Chu *et al.* 2016; He *et al.* 2016), point mutation (Armstrong *et al.* 2016; Wang *et al.* 2016) and replacement (Lin and Potter 2016; Luo *et al.* 2016). It relies on a Cas9/gRNA complex in which the Cas9 protein cleaves at a specific target site under the guidance of a single guide RNA (sgRNA). The cleavage by Cas9 leads to DNA double-strand breaks (DSBs) which could be repaired through either non-homologous end joining (NHEJ) or homology-directed repair (HDR)(Mali *et al.* 2013). The NHEJ repair pathway often generates indels including non-specific insertions or deletions, whereas the HDR pathway induces precise gene editing at the target site taking advantage of homologous templates (Mali *et al.* 2013). In this study, we achieved site-specific *fat-1* insertion in PFFs (Porcine fetal fibroblasts) via CRISPR/Cas9. The single-copy *fat-1* resulted in significant reduction of n-6PUFAs/n-3PUFAs ratio in transgenic pigs, providing a practical reference for further genetic breeding studies.

## Materials and Methods

### Animals

All pigs were obtained from the Huichang Animal Husbandry Science and Technology Co., Ltd. All animal studies were approved by the Animal Welfare and Research Ethics Committee at Jilin University (ratified ID: 20160601), and all procedures were carried out in strict accordance with The Guide for the Care and Use of Laboratory Animals.

### Vector construction

The pROSA26-specific sgRNA was cloned into the PX330 vector (Addgene) to make a functional Cas9/gRNA vector, which is designated as pX330-sgRNA91 hereafter. Pig albumin 5′UTR and 3′UTR synthesized by GENEWIZ (Suzhou, China) were cloned into the psiCheck-2 vector (Promega). The 5′UTR was inserted immediately after the T7 promoter and the 3′UTR was inserted immediately after renilla luciferase gene. The renilla luciferase was reporter gene and the firefly luciferase served as reference gene. We designated this intact vector as psiCheck-2-pig albumin UTR. The codons for the *fat-1* gene from *C. elegans* (GenBank: NM_001028389) were optimized for efficient expression in mammals. The splicing acceptor (SA) sequence, 5′UTR sequence of porcine albumin, optimized *fat-1* sequence, 3′UTR sequence of porcine albumin and SV40 PolyA sequence were then synthesized together by GENEWIZ (Suzhou, China) to constitute the transgene fragment for insertion. Based on our previous work, the donor vector showed a higher knock-in efficiency at the pROSA26 locus when it contained a 5′ HA of approximately 0.5 kb and a 3′ HA of approximately 1.0 kb (Xie *et al.* 2017). Therefore, the optimized HAs were PCR amplified and cloned into the PUC57 vector (Addgene). Finally, the synthesized fragment containing *fat-1* was inserted between the 5′ HA and 3′ HA. We designated this intact targeting vector as PUC57-*fat-1*-KI (Figure 2A).

### Electroporation and luciferase assay

Approximately 30 µg of psiCheck-2-pig albumin UTR plasmid and 30 µg of control psiCheck-2 plasmid were respectively transfected into 3×10^6^ PK-15 cells using the BTX-ECM 2001 Electroporation system. 48 hr later, the cell culture medium was removed and the cells were lysed to detect Firefly and Renilla luminescent according to the protocol of Dual Luciferase Reporter Gene Assay Kit (Beyotime Biotechnology, Shanghai, China) with a Infinite 200 Pro (TECAN).

### Electroporation and selection of PFFs

Approximately 30 µg of pX330-sgRNA91 plasmid and 30 µg of donor puc57-*fat-1*-KI plasmid were cotransfected into 3×10^6^ PFFs. Two days after electroporation, cells were seeded into 100 mm dishes and nine days later individual cell clones were picked and cultured in 24-well plates. When the cells reached approximately 80% confluence, 10% of them were lysed using NP40 lysis buffer (0.45% NP40 plus 0.6% proteinase K). The lysate was used as the PCR template for genotyping. The 1F primer designed in the 5′HA was 5′- GCATTGAGACTGCGTGTTATTAC -3′, and the 1R primer designed in the 3′HA was 5′-ATTCAAAAGACATAAAGGGGAG-3′. The PCR conditions are shown below: 94° for 5 min, followed by 35 cycles of 94° for 30 s, 60° for 30 s, and 72° for 2 min, with a final incubation step at 72° for 5 min. The 2F primer designed outside the 5′HA was 5′-GGTCCCAAATGAGCGAAAC-3′, and the 2R primer designed in the *fat-1* CDS was 5′-TGATGACGCACTGCACTCTTT-3′. The PCR conditions for this primer pair are shown below: 94° for 5 min, followed by 35 cycles of 94° for 30 s, 58° for 30 s, and 72° for 1 min 50 s, with a final incubation step at 72° for 5 min. All PCRs were performed using taq DNA polymerase (TIANGEN). The primer pair 1F/1R was used to identify the insertion of the target gene, and the primer pair 2F/2R was used to identify the site-specific integration of *fat-1*. The PCR amplicons of one individual cell clone were TA cloned using pGM-T Fast Ligation Kit (TIANGEN) and then sequenced. Next, total RNA was extracted from PCR-positive clones using TRNzol-A+ Reagent (TIANGEN) according to the manufacturer’s instructions. An aliquot of 1 μg RNA was used to generate cDNA using a BioRTcDNA First Strand Synthesis Kit (Bioer Technology). The cDNA template was PCR amplified to confirm expression of the *fat-1* gene in positive clones. The following primers were used: 3F: 5′-TGTGTGGATTCAGGACAAGG-3′ and 3R: 5′-CCAGTAGTACCAGAACCAGTTG-3′. The PCR conditions are shown below: 94° for 5 min, followed by 35 cycles of 94° for 30 s, 58° for 30 s, and 72° for 30 s, with a final incubation step at 72° for 5 min.

### SCNT and embryo transfer

The *fat-1*-KI-positive PFF cells cultured in 24-well plates were used to perform SCNT, which was carried out according to previous studies (Lai *et al.* 2002). The positive PFFs were injected into the perivitelline cytoplasm of enucleated oocytes to form reconstructed embryos. The reconstructed embryos were subsequently activated and cultured for approximately 18 h followed by embryo transfer, as described by Lai *et al.* (Yang *et al.* 2011).

### Genotype analysis of the cloned piglets

To confirm insertion and site-specific integration of the *fat-1* gene, genomic DNA extracted from the ears of cloned piglets was used as a template for PCR using the 1F/1R and 2F/2R primer pairs, as described above. Total RNA was extracted from the tails of cloned piglets, and RT-PCR was performed to detect *fat-1* mRNA using the 3F/3R primer. The PCR products were subjected to electrophoresis and sequencing.

### Southern blot analysis

A Southern blot analysis was performed as described previously (Southern 2006). Briefly, approximately 20 μg of high-quality genomic DNA was digested by *Bam*H I and then subjected to agarose gel electrophoresis. Next, the DNA fragments were transferred to a nylon membrane (Amersham). The probe fragment (729bp) was partial of *fat-1* CDS (1212bp) and probe primers specific for *fat-1* CDS were F: 5′-TCAACGCCAACACCAAGCA-3′ and R: 5′-GGTAGGTCACGATCACCAGCAT-3′. The PCR product labeled with digoxigenin by a PCR DIG Probe Synthesis Kit (Roche) were tested by gel electrophoresis and finally the purified probe was hybridized with the DNA fragments on the membrane.

### Karyotype analysis

Porcine tail fibroblasts isolated from newborn piglets were treated with 10 μg/mL demecolcine for 12 h, incubated in 75 mM KCl at 37° for 30 min, fixed on a glass slide using 3:1 methanol/acetic acid for 20 min, stained with Giemsa for 10 min, and finally imaged under the microscope (Nikon eclipse Ti).

### Transcriptional analysis of fat-1 gene in transgenic pigs using real-time PCR

Total RNA from heart, liver, spleen, lung, kidney, skeletal muscle, brain and tongue of *fat-1* knock-in piglet were extracted separately using the TRNzol-A+ reagent (Tiangen, Beijing, China). An aliquot of 1 μg RNA was used to generate cDNA using a BioRTcDNA First Strand Synthesis Kit (Bioer Technology) and the resulting samples were used to perform real-time PCR to quantify *fat-1* expression at the transcriptional level. Porcine GAPDH served as the reference gene. The primers of *fat-1* gene and GAPDH gene were shown as follows: *fat-1* forward (5′-TGTGTGGATTCAGGACAAGG-3′) and reverse (5′-CCAGTAGTACCAGAACCAGTTG-3′), GAPDH forward (5′-GCCATCACCATCTTCCAGG-3′) and reverse (5′-TCACGCCCATCACAAACAT-3′).

### Fatty acid analysis

To evaluate the activity of fatty acid desaturase in *fat-1* transgenic pigs, total muscle fatty acids were extracted as described in previous studies (Han *et al.* 2016b; Lu *et al.* 2008). The fatty acid components were then analyzed by gas chromatography spectrometry (7890A, Agilent Technologies, USA) equipped with a SP-2560 capillary column (100 m*0.25 mm*0.2 µm, Sigma). The initial temperature of the column was maintained at 140° for five min, and then raised to 220° for 40 min at a rate of 4° / min. The percentage of each fatty acid was calculated using a peak area normalization method.

### Statistical analysis

Statistical analysis was performed using a two tailed Student’s *t*-test, and p values < 0.05 were considered significant.

### Data availability

The authors state that all data necessary for confirming the conclusions presented in the article are represented fully within the article.

## Results

### Optimization of fat-1 expression donor vector

**Figure 1:**
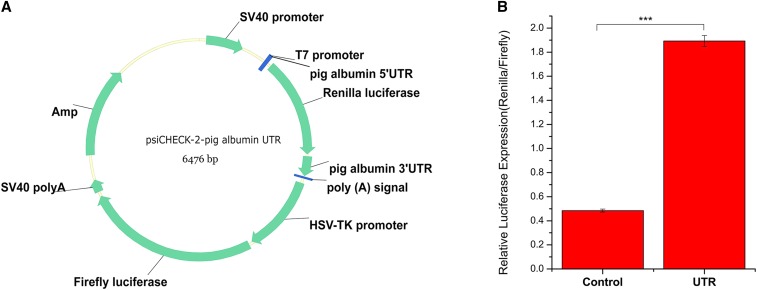
Dual luciferase assays in PK-15 cells. (A) Schematic of the psiCheck-2-pig albumin UTR plasmid; (B) The relative luciferase expression between psiCheck-2-pig albumin UTR plasmid transfected cells and control psiCheck-2 plasmid transfected cells. N = 3, *** *P* < 0.0001.

**Figure 2:**
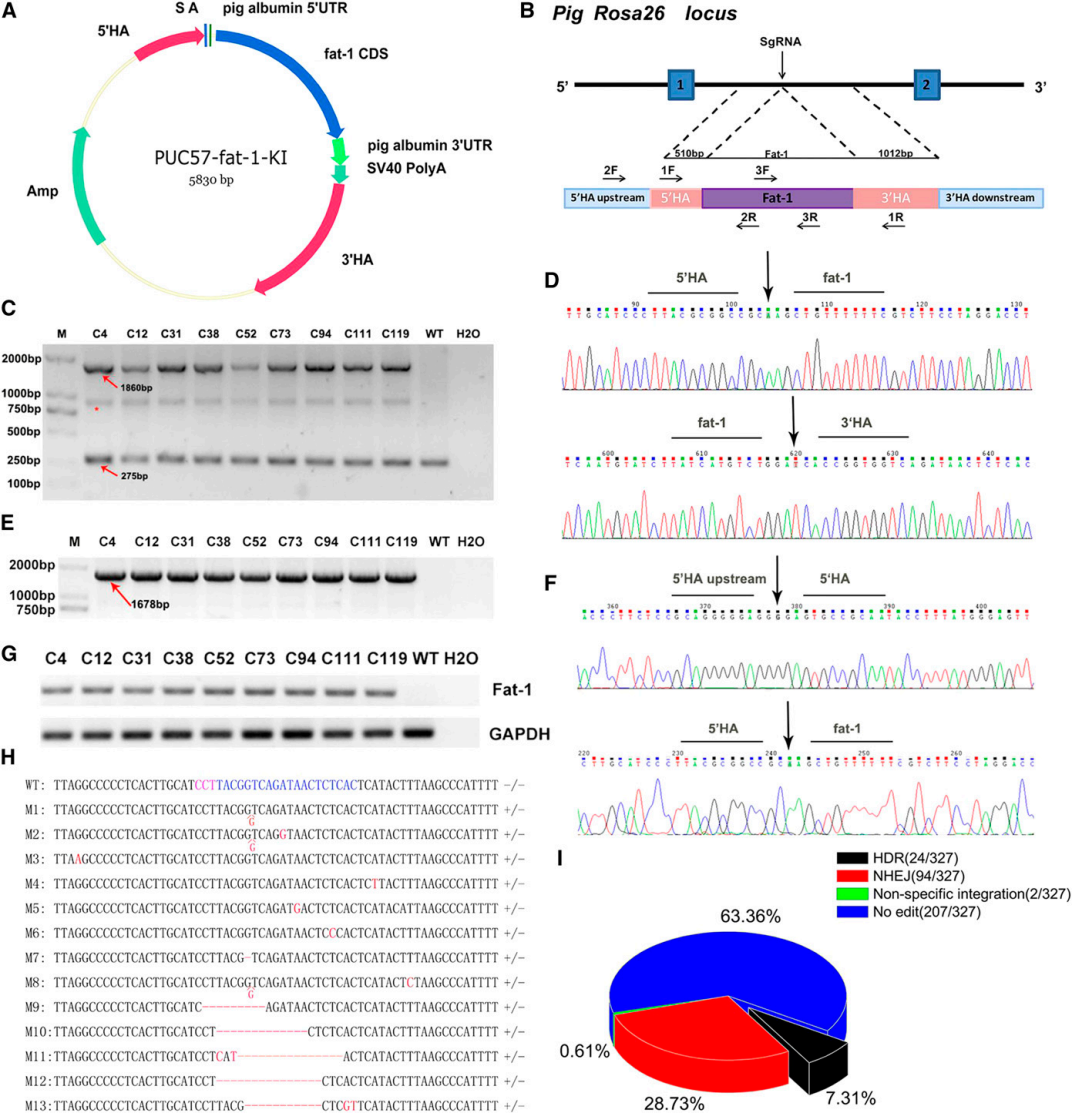
Site-specific *fat-1* knock-in at the pRosa26 locus. (A) Schematic of the donor plasmid for the *fat-1* insertion. 5′HA, 5′ homologous arm (0.5 kb); 3′HA, homologous arm (1.0 kb); (B) Strategy of Cas9-mediated knock-in of *fat-1* into the pRosa26 locus; (C) PCR analysis of the PFF clones using primer pair 1F/1R. The 1F primer was designed in 5′ HA and the 1R primer designed in 3′ HA. M: D2000. WT: negative control. H_2_O: blank control. *: A hybrid band formed by knock-in band and wild-type band in the process of genomic PCR of heterozygous animals; (D) Sanger sequencing results of the PCR products in (C) ; (E) PCR analysis of the PFF clones using primer pair 2F/2R. The 2F primer was designed upstream of 5′ HA and the 2R primer was designed in the *fat-1* CDS; (F) Sanger sequencing results of the PCR products in (E) ; The primer pair 1F/1R was used to identify the insertion of the *fat-1* gene whereas the primer pair 2F/2R was used to identify the site-specific integration of *fat-1*; (G) RT-PCR was performed to confirm the transcription of the *fat-1* gene in genetically positive clones using the 3F/3R primer (268bp) ; (H) The wild-type sequence is placed on the first line and the mutation sequence of single-cell clones were placed below. The PAM was marked in purple and the target site was marked in blue. −/− means wild-type allele and +/− means NHEJ occurred in one allele; (I) The HDR, NHEJ and non-specific integration ratio of 327 individual cell cones.

Serum albumin is the richest protein in mammalian plasma (Bujacz 2012; Nishijima *et al.* 2014). So the 5′UTR and 3′UTR of porcine albumin were thought to have a great potential in regulating gene expression. In our study, the 5′UTR and 3′UTR of porcine albumin were cloned into priCheck-2 and then tansfected into PK-15 cells to verify the up-regulation. Results showed the presence of porcine albumin UTR significantly increased the relative luciferase expression (*P* < 0.0001) (Figure 1). Therefore, the porcine albumin 5′UTR was added in the upstream of *fat-1* CDS and the porcine albumin 3′UTR was added in the downstream of *fat-1* CDS for site specific knock-in (Figure 2 A, B).

### CRISPR/Cas9-mediated integration of fat-1 in PFFs

To insert the *fat-1* CDS at the first intron of the pRosa26 locus, pX330-sgRNA91 and PUC57-*fat-1*-KI plasmids were cotransfected in PFFs by electroporation. As the donor vector for HDR, PUC57-*fat-1*-KI plasmid contained no selectable markers or exogenous promoters (Figure 2A). Instead, the endogenous pRosa26 promoter was utilized to drive the expression of *fat-1*. Two days after electroporation, cells were seeded into 100 mm dishes, and nine days later individual cell clones were collected. PCR products (2F/2R) spanning the junction regions were sequenced to determine the existence of the site-specific insertion of the *fat-1* gene. PCR amplicons (1F/1R) covering the integration site were TA cloned and sequenced to assess the NHEJ events. PCR products (3F/3R) covering a smaller fragment of *fat-1* were used to assess non-specific integration. A total of 24 cell clones out of 327 single-cell clones exhibited the intended bands following both HA-PCR and junction-PCR (Figure 2C, E). The subsequent sequencing results further confirmed the precise integration of *fat-1* at the pRosa26 locus (Figure 2D, F) but all of the positive clones were heterozygous. NHEJ events occurred in 94 individual cell clones including deletions, point mutation and single base insertion (Figure 2H, I and Figure S4 in File S1). The 1F/1R and 3F/3R PCR products of clone No. 74 and clone No. 187 showed expected bands whereas 2F/2R PCR products did not showed expected bands. This result indicated that *fat-1* gene was non-specific integrated into pig genome in clone No. 74 and clone No. 187 (Figures S1–S3 in File S1). Overall, the HDR, NHEJ and non-specific integration ratio was 7.31%, 0.61% and 28.73% respectively (Figure 2I). Finally, total RNA was extracted from these KI clones and the RT-PCR results confirmed transcription of the *fat-1* gene (Figure 2G).

### Generation and genotyping of cloned piglets

**Figure 3:**
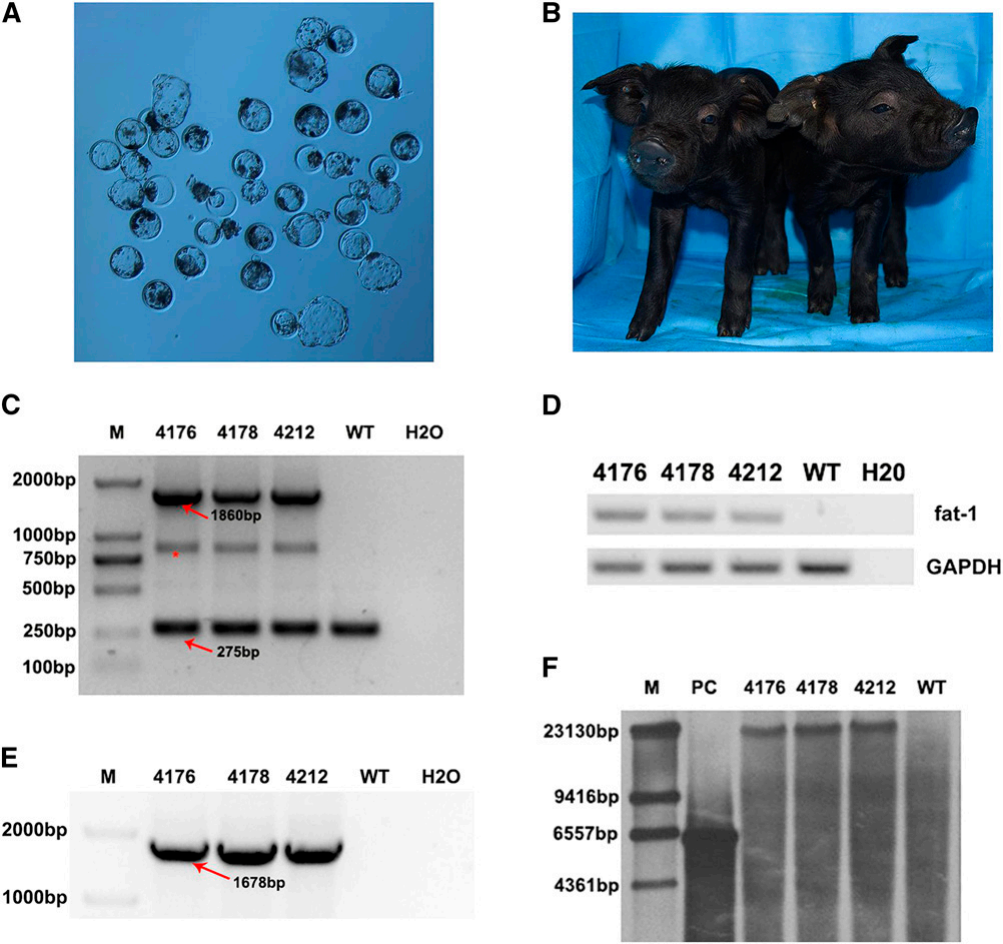
Generation and genotyping of cloned piglets. (A) The reconstructed embryos were cultured *in vitro* for approximately 6 days until the blastocyst stage; (B) Photo of site-specific *fat-1* knock-in piglets at seven days after birth; (C, E) PCR analysis of *fat-1* knock-in pigs using primer 1F/1R and primer 2F/2R respectively. *: A hybrid band formed by knock-in band and wild-type band in the process of genomic PCR of heterozygous animals; (D) RT-PCR analysis of *fat-1* positive pigs using primer 3F/3R; (F) The Southern blot result of *fat-1* knock-in pigs using the digoxigenin labled *fat-1* specific probe.

Prior to the reconstructed embryo transfer, wild-type and *fat-1* knock-in PFFs were used as donor cells to perform SCNT and examine the developmental potency of reconstructed embryos. Our results demonstrated that wild type and *fat-1* knock in PFFs donor cells shared a similar rate of blastocyst development (22.57 ± 0.1122% *vs.* 22.44 ± 0.2323%, *P* > 0.05, n = 3) (Figure 3A and Table S1 in File S1) Hence, the *fat-1* gene insertion has no adverse effects on the development of reconstructed embryos. A total of 500 reconstructed embryos were surgically transferred to the uterus of five recipients, and two recipients were pregnant (Table S2 in File S1). After approximately 114 days of gestation, three piglets were born by eutocia with an average birth weight of 1.17 kg (Figure 3B). All of them grew and developed normally. The PCR and sequencing results showed that all piglets contained the site-specific *fat-1* knock-in at the pRosa26 locus (Figure 3C, D); RT-PCR also confirmed the expression of *fat-1* in the tails of these cloned pigs. Chromosome karyotype analysis showed that the positive pigs had normal chromosome numbers (Figure S5 in File S1). Furthermore, Southern blotting results showed an intended band, indicating a single insertion site for *fat-1* in the porcine genome (Figure 3F). In summary, we obtained cloned pigs that harbored a site-specific integration of *fat-1* at the pRosa26 locus.

### F1 generation of fat-1 knock-in pigs and transcriptional analysis of fat-1 gene

**Figure 4:**
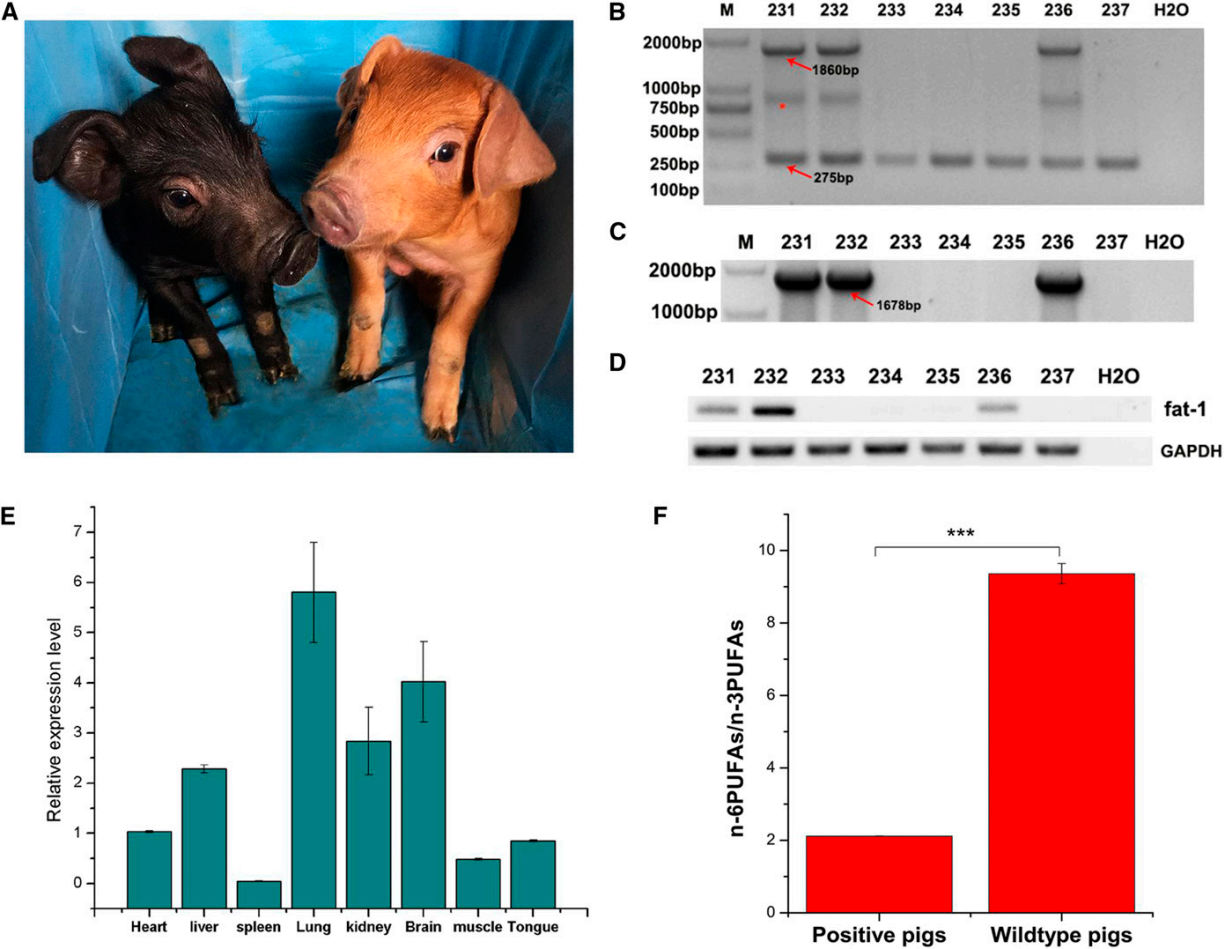
Transcriptional analysis of *fat-1* gene and fatty acid analysis of muscle tissues of F1 generation. (A) Photo of F1 generation *fat-1* knock-in piglets at ten days after birth; The F1 generation was the offspring of Songliao Black sow and Duroc boars. (B, C) PCR analysis of F1 generation using primer 1F/1R and primer 2F/2R. *: A hybrid band formed by knock-in band and wild-type band in the process of genomic PCR of heterozygous animals; (D) RT-PCR analysis of F1 generation using primer 3F/3R; (E) Transcriptional level analysis of *fat-1* gene in a wide variety of tissues as determined by real-time PCR; (F) The n-6PUFAs/n-3PUFAs ratio of *fat-1* knock-in pigs and wild-type pigs. There are seven F1 generations and three of them were *fat-1* knock-in pigs. The three positive piglets and three littermate wild-type piglets were killed and muscle fatty acids were extracted to evaluate the n-6PUFAs/n-3PUFAs ratio. Values are denoted as the Mean± SEM, n = 3, *** *P* < 0.0001.

One of the *fat-1* knock-in pigs was mated with Duroc boars, and 7 F1 generation piglets were born (Figure 4A). Three piglets are heterozygous for *fat-1*gene insertion at the pRosa26 locus as evidenced by RT-PCR and sequencing (Figure 4B, C and D). To quantify *fat-1* expression at the transcriptional level, total RNA from the heart, liver, spleen, lung, kidney, skeletal muscle, brain and tongue of *fat-1* knock-in piglets were subjected to real-time PCR. Results show that *fat-1* gene expressed in a wide variety of tissues and has the highest expression in the lung whereas the lowest expression in the spleen among all the tissues detected. In addition, the *fat-1* expression level in the kidney and brain was also relatively high (Figure 4E).

### Analysis of fatty acids in the fat-1 knock-in pigs

To further determine whether the *fat-1* knock-in pigs exhibit a beneficial phenotype, fatty acids extracted from the muscle tissue of *fat-1* and wild-type pigs were measured using gas chromatography. The level of LA and AA in *fat-1* pigs decreased dramatically compared with wild-type pigs. In contrast, the level of n-3PUFAs including ALA, DHA and DPA in *fat-1* pigs were significantly higher than those in wild-type pigs, leading to an obvious decrease in the n-6PUFAs/n-3PUFAs ratio of *fat-1* pigs from 9.36 to 2.12 (Figure 4F and Table 1). Collectively, these results demonstrate that the single copy knock-in of *fat-1* driven by Rosa26 promoter is sufficient to convert n-6PUFAs to n-3PUFAs so as to reduce the n-6PUFAs/n-3PUFAs ratio in these pigs.

**Table 1:** Quantification of PUFAs in *fat-1* knock-in and wild-type pigs

Fatty acids	*Fat-1* knock-in pigs	Wild-type pigs
n-6 PUFAs		
C18:2(LA)	4.719 ± 0.04711***	7.781 ± 0.002225
C20:4(AA)	3.374 ± 0.03778***	4.518 ± 0.01559
Total n6	8.094 ± 0.01791***	12.30 ± 0.01623
n-3 PUFAs	0.4084 ± 0.005407***	0.2510 ± 0.002812
C18:3(ALA)	0.06893 ± 0.008027	0.08027 ± 0.00256
C20:5(EPA)	0.6380 ± 0.007916***	0.5159 ± 0.006721
C22:6(DHA)	2.693 ± 0.01361***	0.4692 ± 0.03188
C22:5(DPA)	3.817 ± 0.008717***	1.316 ± 0.03965
Total n3	2.120 ± 0.00111***	9.360 ± 0.2790
n-6/n-3 ratio		

Each fatty acid is presented as the percentage in total fatty acid. Values are denoted as the Mean± SEM, n = 3, *** *P* < 0.0001.

## Discussion

N-3PUFAs mainly derived from marine products have beneficial health effects related to many diseases such as cardiovascular diseases, inflammatory disease and cancer. As the most eaten meat in the world, pork can be an important source of n-3PUFAs especially for populations that barely consumed marine products (Dugan *et al.* 2015). But high n-6PUFAs/n-3PUFAs ratio of regular pork makes it a poor source of n-3PUFAs. Transgenic technologies give us a new sight to solve the problem and thus n-3PUFAs-rich transgenic pigs were generated. In the studies of Lai *et al.*, the concentration of n-3PUFAs in *fat-1* transgenic pigs tails were significantly increased, leading to a drop in the n-6PUFAs/ n-3PUFAs ratio from 8.52 to 1.69 (Lai *et al.* 2006). Zhang *et al.* reported that the ratio of n-6PUFAs/n-3PUFAs in the *fat-1* transgenic pig muscle decreased from 48.85 to 10.91 (Zhang *et al.* 2012). However, all previous *fat-1* transgenic pigs were generated using random integration which introduced the exogenous CAG promoter and selectable marker genes. Although the CAG promoter is a powerful synthetic promoter constantly used to induce the high level of gene expression in mammals (Sakaguchi *et al.* 2014), the methylation of it often leads to unstable expression (Zhou *et al.* 2014) even silence of transgenes (Duan *et al.* 2012). The introduction of selectable marker genes would increase public concern on biosafety of transgenic animals, thus limiting the commercial application. Therefore, it is essential to generate site-specific integration as well as selectable marker-free transgenic animals. In our study, we implemented precise genome editing in PFFs, whereby *fat-1* was integrated in a site-specific fashion to the pRosa26 locus based on CRISPR/cas9-mediated HDR. Three heterozygous *fat-1* knock-in pigs were obtained via SCNT. The Southern blot result indicated no random integration was found in any of the three positive pigs. Furthermore, karyotype analysis indicated they had normal chromosome numbers (Figure S5 in File S1). Additionally, we obtained three F1 piglets in which the *fat-1*gene was inserted at pRosa26 locus and expressed in a wide variety of tissues. Based on our fatty acid analysis of F1 muscle tissue, the proportion of LA dropped by 1.65 times in *fat-1* pigs compared with wild-type pigs and the AA dropped by 1.34 times. The proportion of n-3PUFAs including ALA, DHA and DPA have risen by 1.63 times, 1.24 times and 5.74 times in the *fat-1* pigs, respectively. As a result, the ratio of n-6PUFAs/n-3PUFAs in *fat-1* pigs decreased dramatically from 9.36 to 2.12.

In summary, we successfully generated site-specific *fat-1* transgenic pigs whose genotype could be transmitted to the next generation and enables obvious decrease of n-6PUFAs/n-3PUFAs with a single-copy of *fat-1* gene. Importantly, this kind of n-3PUFA-rich pork would bring health benefits to consumers compared to regular pork products. At the same time, the site-specific *fat-1* transgenic pigs can serve as an animal model to investigate therapeutic effects of n-3PUFAs on various diseases.

## Supplementary Material

Supplemental Material is available online at www.g3journal.org/lookup/suppl/doi:10.1534/g3.118.200114/-/DC1.
