# Supplementary material for: Site-Specific Fat-1 Knock-In Enables Significant Decrease of n-6PUFAs/n-3PUFAs Ratio in Pigs
Source: G3 (Bethesda). 2018 Mar 21;8(5):1747–54. doi: 10.1534/g3.118.200114 (PMC5940165; doi:10.1534/g3.118.200114)
Supplement: Supplementary file 1 [file 1747FileS1.doc]

# Site-specific Fat-1 Knock-in enables significant decrease of n-6PUFAs/n-3PUFAs ratio in pigs

Mengjing Li^1‡^, Hongsheng Ouyang^1‡^, Hongming Yuan^1^, Zicong Xie^1^, Kankan Wang^1^, Tingting Yu^1^, Minghao Liu^1^, Xue Chen^1^, Xiaochun Tang^1^, Huping Jiao^1^, Daxin Pang^1^*

Jilin Provincial Key Laboratory of Animal Embryo Engineering, College of Animal Sciences, Jilin University, Changchun, Jilin Province, People’s Republic of China

‡These authors contributed equally to this work.

**Fig.S1**

**
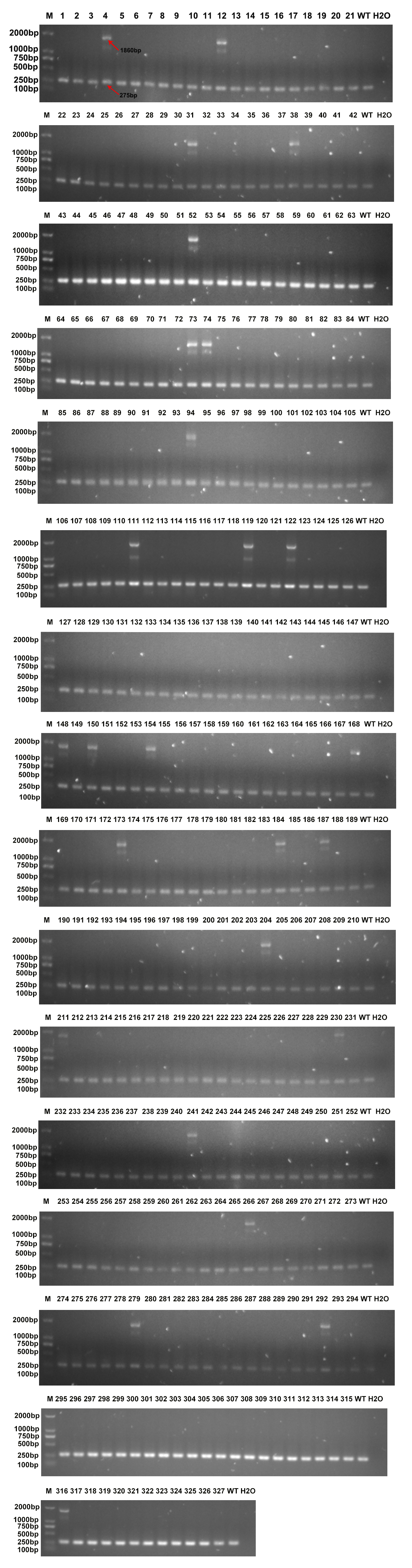
**

**Figure S1:** The agarose gel electrophoresis of 327 individual cell clones using primer 1F/1R.

**Fig.S2**

**
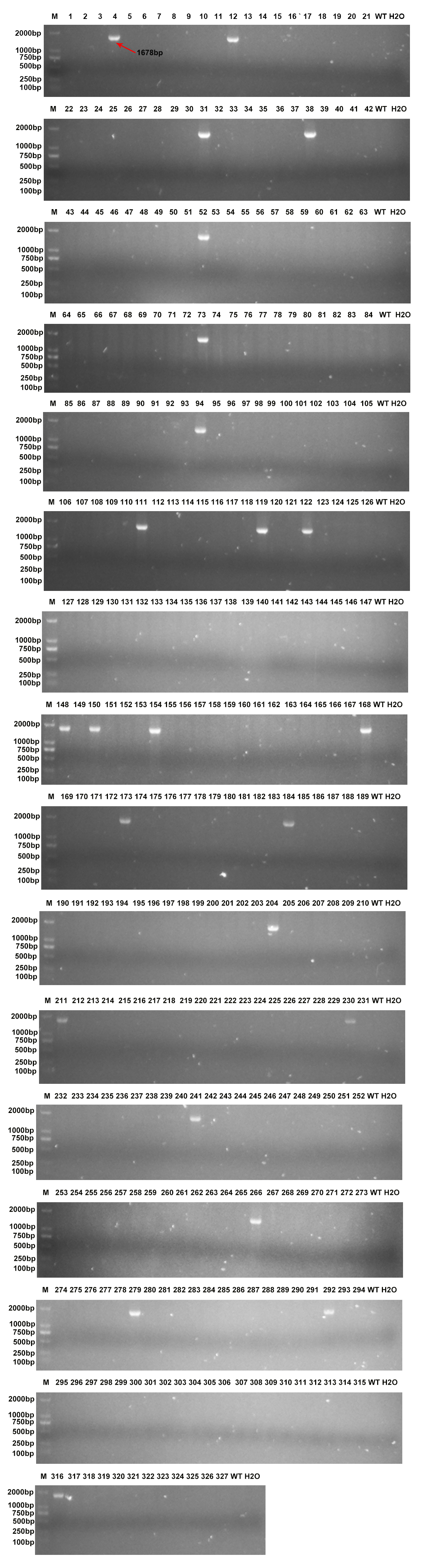
**

**Figure S2:** The agarose gel electrophoresis of 327 individual cell clones using primer 2F/2R.

**Fig.S3**

**
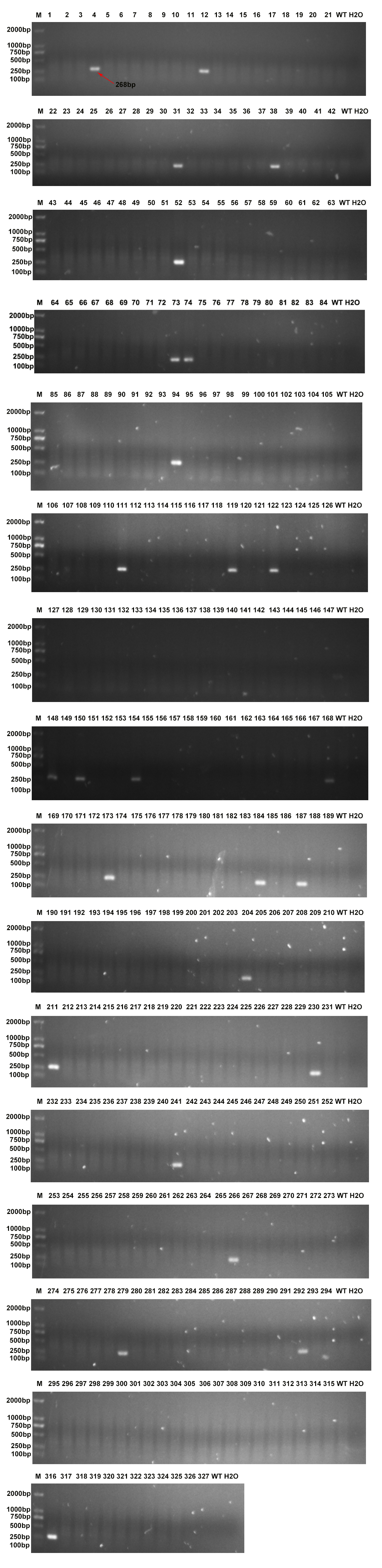
**

**Figure S3:** The agarose gel electrophoresis of 327 individual cell clones using primer 3F/3R.

**Fig.S4**

**
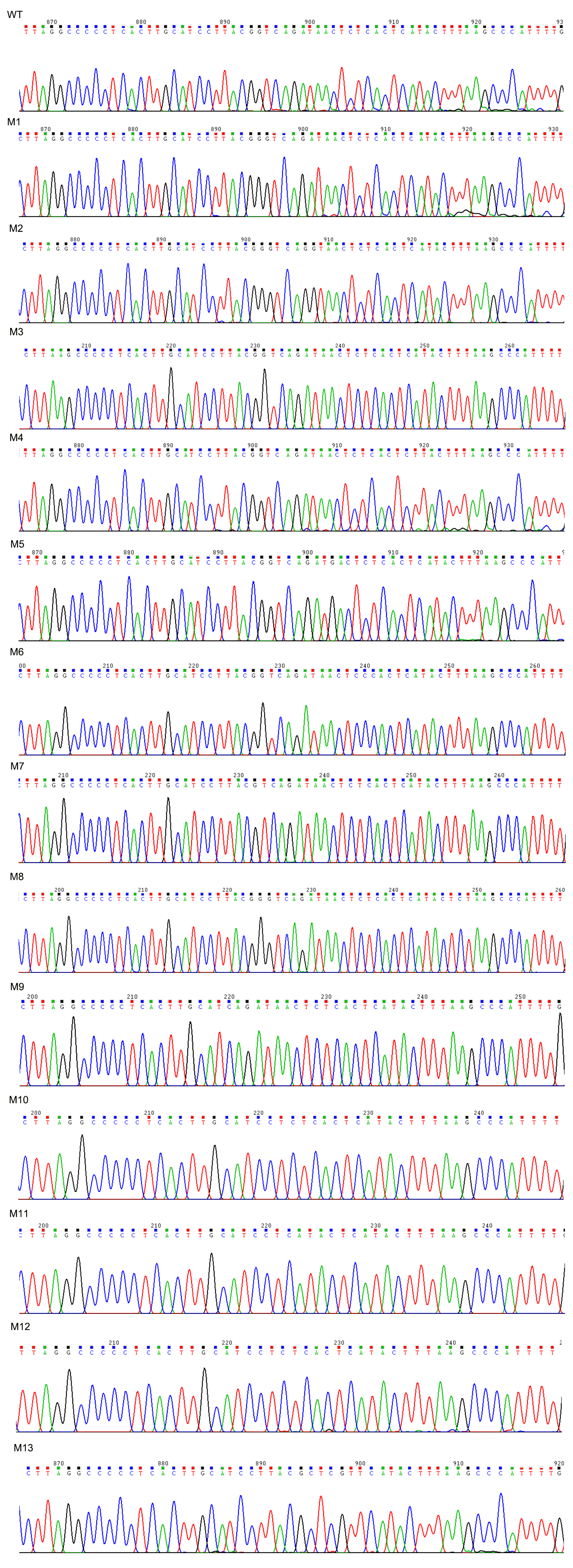
**

**Figure S4:** The Sanger sequencing results of PCR products harboring targeting sites from mutant individual cell clones.

**Fig.S5**

**
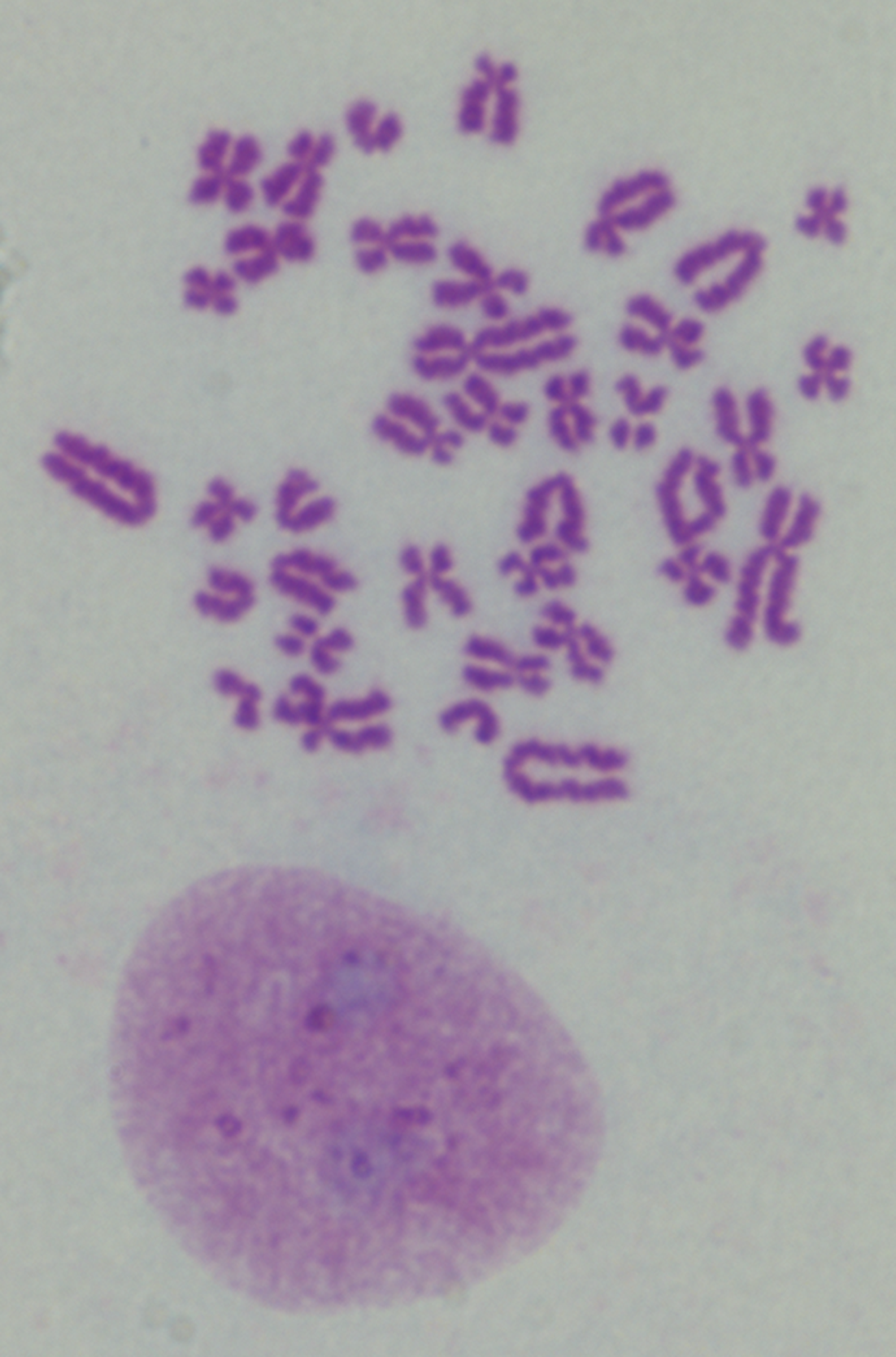
**

**Figure S5:** The karyotype analysis showed a normal chromosomal number in *fat-1* knock-in pigs.

Sequences used in this study:

1) The *fat-1* expression donor sequence. The sequence marked in dark was 5' HA. The sequence marked in yellow was SA. The sequence marked in green was pig albumin 5'UTR. The sequence marked in red was *fat-1* CDS. The sequence marked in blue was pig albumin 3'UTR. The sequence marked in dark red was SV40 PolyA. The sequence marked in orange was 3' HA.

ggagtgccgcaatacctttatgggagttctctgctgcctccttttcctaaggaccgccctgggcctagaaaaatccctccctcccccgcgatctcgtcatcgcctccatgtcagtttgctccttctcgattatgggcgggattcttttgccctggcttaacctgattcttgggcgttgtcctgcaggggattgagcaggtgtacgaggacgagcccaatttctctatattcccacagtcttgagtttgtgtcacaaaataattatagtggggtggagatgggaaatgagtccaggcaacacctaagcctgattttatgcattgagactgcgtgttattactaaagatctttgtgtcgcaatttcctgatgaagggagataggttaaaaagcacggatctactgagttttacagtcatcccatttgtagacttttgctacaccaccaaagtatagcatctgagattaaatattaatctccaaaccttaggccccctcacttgcatccttacaagctgttttttcgtcttcctaggaccttttctcttctatcaaccccacaagcctttggcacaatggtcgcccacagcagcgagggcctgagcgccaccgcccctgtgacaggcggcgacgtgctggtcgacgccagagccagcctggaggagaaggaggcccccagggacgtcaacgccaacaccaagcaggccaccaccgaggagcccagaatccagctgcccaccgtggacgccttccggagagccatccccgcccactgcttcgagcgggacctggtgaagagcatccgctacctggtgcaggacttcgccgccctgaccatcctgtacttcgccctgcccgccttcgagtacttcggcctgttcggctatctggtgtggaacatcttcatgggcgtgttcggcttcgccctgttcgtggtgggccacgactgcctgcacggcagcttcagcgacaaccagaacctgaacgacttcatcggccacatcgccttcagccccctgttcagcccctacttcccctggcagaagagccacaagctgcaccacgccttcaccaaccacatcgacaaggaccacggccatgtgtggattcaggacaaggactgggaggccatgcccagctggaagcggtggttcaaccccatccccttcagcggctggctgaagtggttccccgtgtataccctgtttggcttctgcgacggcagccacttctggccttacagcagcctgtttgtgcggaacagcgaaagagtgcagtgcgtcatcagcggcatctgctgctgtgtgtgcgcctacatcgccctgacaatcgccggcagctacagcaactggttctggtactactgggtgcccctgagcttcttcggcctgatgctggtgatcgtgacctacctgcagcacgtggacgacgtggccgaagtgtacgaggccgacgagtggagctttgtgcggggccagacccagaccatcgaccgctactacggcctgggcctggacaccaccatgcaccacatcaccgacggacacgtggcccatcactttttcaacaagatcccccactaccacctgatcgaggccaccgagggcgtgaagaaagtgctggagcccctgagcgacacccagtacggctacaagagccaggtgaactacgacttcttcgcccggttcctgtggttcaactacaagctggactatctggtgcacaagaccgccggcatcatgcagttccggaccaccctggaggaaaaggccaaggccaagtgatgaacaacacagtgacaagcatctcagactaccctgagaataagagaaagagaaatgaagacctagacttatccatctctttttcttttctgttggttttaaaccaacaccctgtctaaagtacacaaatttctttaaatattttgcctcttttctctgtgctacaattaataaaaaaatgaaaagaatctttgtttattgcagcttataatggttacaaataaagcaatagcatcacaaatttcacaaataaagcatttttttcactgcattctagttgtggtttgtccaaactcatcaatgtatcttatcatgtctggatcggtcagataactctcactcatactttaagcccattttgtttgttgtacttgctcatccagtcccagacatagcattggctttctcctcacctgttttaggtagccagcaagtcatgaaatcagataagttccaccaccaattaacactacccatcttgagcataggcccaacagtgcatttattcctcatttactgatgttcgtgaatatttaccttgattttcatttttttctttttcttaagctgggattttactcctgaccctattcacagtcagatgatcttgactaccactgcgattggacctgaggttcagcaatactcccctttatgtcttttgaatacttttcaataaatctgtttgtattttcattagttagtaactgagctcagttgccgtaatgctaatagcttccaaactagtgtctctgtctccagtatctgataaatcttaggtgttgctgggacagttgtcctaaaattaagataaagcatgaaaataactgacacaactccattactggctcctaactacttaaacaatgcattctatcttcacaaatgtgaaaaaggagttccctcagtggactaaccttatcttttctcaacacctttttctttgcacaattttccacacatgcctacaaaaagtacttttctgctcaagtcacactgagttgattgctatttaccaaaatcaaagtaacattatcagatctctgtagggtggttccctctggaatgctaccctccatagtccttacccttcaagtaaagagcatgaagactgaaatatctcctctgtgatctgtcatcctttaagccagaatcccccataaaaaagttagtattgctttctcctgatcccatagcaggttgaatcatagcacttatcaggttgttgtcattgcttgcttaaattctcctaactatttggagcttcttgagggcacaggttcttgttgagtcttgtacctaagcacctagtatagtccttgatgtctagccaac

2) The probe sequence for *fat-1* gene

tcaacgccaacaccaagcaggccaccaccgaggagcccagaatccagctgcccaccgtggacgccttccggagagccatccccgcccactgcttcgagcgggacctggtgaagagcatccgctacctggtgcaggacttcgccgccctgaccatcctgtacttcgccctgcccgccttcgagtacttcggcctgttcggctatctggtgtggaacatcttcatgggcgtgttcggcttcgccctgttcgtggtgggccacgactgcctgcacggcagcttcagcgacaaccagaacctgaacgacttcatcggccacatcgccttcagccccctgttcagcccctacttcccctggcagaagagccacaagctgcaccacgccttcaccaaccacatcgacaaggaccacggccatgtgtggattcaggacaaggactgggaggccatgcccagctggaagcggtggttcaaccccatccccttcagcggctggctgaagtggttccccgtgtataccctgtttggcttctgcgacggcagccacttctggccttacagcagcctgtttgtgcggaacagcgaaagagtgcagtgcgtcatcagcggcatctgctgctgtgtgtgcgcctacatcgccctgacaatcgccggcagctacagcaactggttctggtactactgggtgcccctgagcttcttcggcctgatgctggtgatcgtgacctacc

**TableS1: In vitro development of reconstructed embryos**

| Donor Cells | No. oocytes used for SCNT | No. reconstructed embryos | No. developed to blastocysts | blastocyst rate |
| --- | --- | --- | --- | --- |
| Fat-1KI-clone-C4 | 370 | 310 | 69 | 22.2% |
| Fat-1KI-clone-C12 | 320 | 280 | 62 | 22.14% |
| Fat-1KI-clone-C31 | 350 | 300 | 70 | 23.33% |
| Wild-type PFFs-C1 | 320 | 290 | 63 | 21.72% |
| Wild-type PFFs-C2 | 380 | 320 | 76 | 23.75% |
| Wild-type PFFs-C3 | 330 | 280 | 61 | 21.78% |

**TableS2. SCNT for the generation of fat-1 knock-in pigs**

| Donor cells | Transferred embryos | Pregnancy | Number of birth | Number of positive piglets |
| --- | --- | --- | --- | --- |
| C38,C52,C73,C94 | 105 | No | 0 | 0 |
| C111,C119,C122,C147 | 102 | Yes | 1 | 1 |
| C150,C154,C168,C173 | 103 | Yes | 2 | 2 |
| C184,C204,C211,C230, | 102 | No | 0 | 0 |
| C241,C266,C279,C292 | 107 | No | 0 | 0 |
